# Effect of Oxytocin on Hunger Discrimination

**DOI:** 10.3389/fendo.2019.00297

**Published:** 2019-05-15

**Authors:** Mitchell A. Head, David C. Jewett, Sarah N. Gartner, Anica Klockars, Allen S. Levine, Pawel K. Olszewski

**Affiliations:** ^1^Faculty of Science and Engineering, University of Waikato, Hamilton, New Zealand; ^2^Department of Psychology, University of Wisconsin-Eau Claire, Eau Claire, WI, United States; ^3^Department of Food Science and Nutrition, University of Minnesota, St. Paul, MN, United States

**Keywords:** oxytocin, satiety, hunger, feeding, hypothalamus

## Abstract

Centrally and peripherally administered oxytocin (OT) decreases food intake and activation of the endogenous OT systems, which is associated with termination of feeding. Evidence gathered thus far points to OT as a facilitator of early satiation, a peptide that reduces the need for a meal that has already begun. It is not known, however, whether OT can diminish a feeling of hunger, thereby decreasing a perceived need to seek calories. Therefore, in the current project, we first confirmed that intraperitoneal (i.p.) OT at 0.3–1 mg/kg reduces food intake in deprived and non-deprived rats. We then used those OT doses in a unique hunger discrimination protocol. First, rats were trained to discriminate between 22- and 2-h food deprivation (hungry vs. sated state) in a two-lever operant procedure. After rats acquired the discrimination, they were food-restricted for 22 h and given i.p. OT before a generalization test session. OT did not decrease 22-h deprivation-appropriate responding to match that following 2-h food deprivation, thus, it did not reduce the perceived level of hunger. In order to better understand the mechanisms behind this ineffectiveness of OT, we used c-Fos immunohistochemistry to determine whether i.p. OT activates a different subset of feeding-related brain sites under 22- vs. 2-h deprivation. We found that in sated animals, OT induces c-Fos changes in a broader network of hypothalamic and brain stem sites compared to those affected in the hungry state. Finally, by employing qPCR analysis, we asked whether food deprivation vs. sated state have an impact on OT receptor expression in the brain stem, a CNS “entry” region for peripheral OT. Fasted animals had significantly lower OT receptor mRNA levels than their *ad libitum*-fed counterparts. We conclude that OT does not diminish a feeling of hunger before a start of a meal. Instead OT's anorexigenic properties are manifested once consumption has already begun which is—at least to some extent—driven by changes in brain responsiveness to OT treatment in the hungry vs. fed state. OT should be viewed as a mediator of early satiation rather than as a molecule that diminishes perceived hunger.

## Introduction

A nine amino acid neuropeptide, oxytocin (OT), synthesized primarily in the hypothalamic paraventricular (PVN) and supraoptic (SON) nuclei, released throughout the CNS and, via the neurohypophysis, into general circulation, has been known to regulate a number of functions, including parturition, lactation, and social behaviors. In 1989, Arletti et al. reported for the first time that intracerebral and intraperitoneal (i.p.) administration of OT in rats causes a marked reduction in food intake (1). Since then, a plethora of evidence has emerged that supports the involvement of OT in termination of feeding.

Injections of OT in the third and fourth cerebral ventricles as well as in numerous brain areas, including the hypothalamic ventromedial nucleus (VMH), dorsal vagal complex, central (CEA), and basolateral (BLA) amygdala, ventral tegmental area (VTA) and nucleus accumbens core (AcbC), and the limbic system, produce cessation of ingestive behavior (2–10). This early termination of feeding after OT treatment pertains to relatively bland laboratory chow, as well as those that are highly palatable (2, 3, 10, 11). Despite its limited ability to cross the blood-brain barrier (BBB), peripherally administered OT (including via intraperitoneal (i.p.), subcutaneous, and intravenous routes) also potently decreases food intake, most likely by engaging the brainstem where the BBB protection is weak (1, 2, 9, 12–14).

Increased c-Fos immunoreactivity in OT neurons and elevated OT plasma levels coincide with meal cessation (15). Hypothalamic OT mRNA expression is downregulated during fasting and restored by re-feeding (16). Administration of molecules that support satiation, such as cholecystokinin (CCK), alpha-melanocyte stimulating hormone, and glucagon-like peptide-1 (GLP-1), increases activity of the OT system (17–21). Furthermore, OT release has been associated with peripheral changes that typically occur as ingestive behavior nears its end, such as elevated osmolality, an increase in select nutrient levels, and excessive stomach distension (9, 22–25).

Overall, those findings strongly point to the role of OT in promoting satiety, facilitating early termination of consumption, and reducing meal size. On the other hand, one aspect of OT's involvement in feeding control that has not been investigated in detail is whether OT has a capacity to reduce a feeling of hunger. That, e.g., peripherally administered OT increases latency to begin a meal, might suggest it to some extent, but direct evidence is lacking. Scarcity of data also stems from methodological difficulties in assessing hunger in laboratory animals.

There is a unique protocol, however, that relies on rats reporting their hunger status using an operant behavior. Animals are trained to discriminate between acute food deprivation lasting 22 h (hunger) or 2 h (no actual energy depletion). In one such study, Corwin et al. trained rats maintained at 80% of their free-feeding body weight to discriminate between food consumed 22 or 3 h before experimental sessions (26). The anorexigen CCK or ingestion of sweetened condensed milk induced effects similar to chow consumption occurring 3 h before a test session, in contrast to fenfluramine which did not reliably produce effects similar to 3-h food ingestion. Thus, CCK produced effects that resembled a lack of hunger. Similar outcomes to those induced by CCK were seen in response to an anti-obesity drug, sibutramine (27, 28).

In the current project, we employed this unique hunger discrimination protocol (employing 22-h vs. 2-h deprivation) to examine whether OT administered i.p. prior to discrimination testing in rats reduces a feeling of hunger. We used OT doses based on previous reports and on the results of two additional feeding studies in hungry and sated animals performed here. In order to better understand whether i.p. OT activates a different subset of feeding-related brain sites depending on the lack of access to food for 22 or 2 h, we conducted an analysis of c-Fos immunoreactivity. Finally, since i.p. OT is thought to directly act at the brain stem, we asked whether food deprivation vs. a sated state has an impact on OT receptor expression established with real-time PCR.

## Materials and Methods

### Animals

Male Sprague-Dawley rats aged 12-weeks old (average b. wt. 400 g) were housed individually in standard plastic cages with wire tops in a temperature-controlled (22°C) animal facility with a 12:12 light:dark cycle (lights on at 08:30 in the discrimination studies and 07:00 in the remaining experiments). Water and standard laboratory chow were available *ad libitum* unless stated otherwise. Animals were treated in accordance with the National Institute of Health Guide for the Care and Use of Laboratory Animals. The University of Waikato Animal Ethics Committee and the University of Wisconsin-Eau Claire Institutional Animal Care and Use Committee approved all procedures described here.

### Behavioral Studies

#### Establishing Effective Doses of OT That Reduce Feeding in Animals Deprived for 22 h and 2 h

As we sought to investigate effects of OT on consumption of the same kind of diet in both 22-h deprived (thus, driven to eat by energy needs) and 2-h deprived (thus, not motivated to eat by energy needs) animals, we chose to give them episodic access to palatable high-fat high-sugar (HFHS; Research Diets) chow. Rodents avidly consume HFHS diet even in the absence of hunger. This was done to test whether i.p. OT retained its anorexigenic properties in the context of 2 and 22 h deprivation scenarios as they were slightly different from previously described protocols that typically relied either on depriving animals overnight, 16 or 24 h (hunger) or on giving animals episodic 2–4 h access to palatable food without depriving them even for 2 h (relative satiety).

##### Effect of OT on palatable food intake in rats deprived for 2 h

In order to examine whether OT decreases the consumption of HFHS food, we adjusted our previously published protocol (14, 29). Rats maintained on *ad libitum* food and water had standard chow taken away at 10:00 (water remained in the cage). Two hour later, HFHS chow was placed in the cages for 2 h. Fifteen minutes prior to the HFHS food presentation, animals received an i.p. injection of isotonic saline or 0.1, 0.3, or 1 mg/kg OT Sigma, St. Louis, MO, USA (*n* = 9/group). The animals had had previous episodic (2 h per day, 5 and 10 days before the study) exposure to this HFHS chow to avoid neophobia. Data were analyzed with a one-way ANOVA followed by Dunnet's *post-hoc* analysis, with a significance level set at *p* ≤ 0.05.

##### Effect of OT on palatable food intake in rats deprived for 22 h

The cohort of rats used in the previous experiment (2.2.1.1) was studied here. A 2-week “washout” period elapsed between the experiments. Animals that had had access to standard chow were food-deprived for 22 h (deprivation ending at 12:00). They were then given access for 4 h to the HFHS chow. Fifteen minutes prior to the HFHS food presentation, animals received an i.p. injection of isotonic saline or 0.1, 0.3, or 1 mg/kg OT. HFHS chow intake after 22 h of deprivation was measured at 1 and 4 h. Data were analyzed with a one-way ANOVA followed by Dunnet's *post-hoc* analysis, with a significance level set at *p* ≤ 0.05.

#### Establishing Effects of OT on Discrimination Between 22 and 2 h of Food Deprivation

Experimentally naïve male Sprague Dawley rats (Harlan, Madison, WI) were ~12-weeks old at the beginning of the procedures. Food (Harlan Teklad chow, Madison, WI) and water were continuously available unless otherwise stated.

##### Apparatus

Daily discrimination sessions were conducted in standard operant chambers equipped with two response levers (Med-Associates, St. Albans, VT), placed in ventilated, sound-attenuating cubicles. Forty-five mg food pellets (Bio-Serve F#0021, Frenchtown, NJ) reinforced lever pressing and were delivered by a pellet dispenser into a food pellet trough located between the two levers. A house light in the back wall of the operant chamber illuminated the chambers during sessions. Experimental contingencies and data recording were performed with Med Associates software and a computer located in an adjacent room.

##### Discrimination training

Rats were initially food deprived to ~85% of their free feeding body weight and trained to press a level via the method of successive approximations. First, a single lever press was reinforced with a 45 mg food pellet (Bio-Serv F#0021, Frenchtown NJ). Response requirements were increased gradually until 15 presses (fixed ratio (FR) 15) were needed to generate food. When reliable responding to both levers was achieved, rats were given free access to food for 305 days before subsequent discrimination training began. Rats were trained to discriminate between 22 and 2 h of acute food deprivation using multiple cycle training. Under 22-h conditions, food was removed 22 h before the training session. Rats were placed into the operant chamber 5 min before the first training cycle. When the first training cycle started, the house light was turned on and 15 left lever presses were reinforced with 45 mg food pellet delivery (FR 15 reinforcement schedule). Incorrect (right) lever presses were punished with 8 s of darkness under an FR 15 schedule. Training continued until 5 reinforcers were earned or 5 min elapsed. At least one more additional training cycle, identical to the first, was conducted 30–120 min after the previous cycle. Under 2-h conditions, the contingencies were reversed: right lever presses were reinforced and left lever presses were punished under the FR 15 schedule. Conditions were quasi-random with the provision that the same training condition (22 or 2 h of food deprivation) could not be given for more than two consecutive sessions.

Discrimination training continued until the subject emitted 80% or greater condition-appropriate responses prior to delivery of the first reinforcer and for the entire training session during all training cycles for 8 of 10 consecutive daily sessions.

##### Generalization test: evaluation of the ability of OT to reduce the discriminative stimulus effects of 22-h food deprivation

The final discrimination tests assessed the ability of OT to reduce the discriminative stimulus effects of 22-h food deprivation. These tests were conducted under 22-h deprivation conditions. During the first response period, only left lever presses were reinforced. Following the first response period, rats were injected i.p. with isotonic saline or OT (0.01–1 mg/kg range). After injections, rats were placed in stainless steel cages without food or water. During the next response period occurring 30 min after the injection, responses toward both levers were reinforced. Generalization tests lasted until the subject earned 5 reinforcers or until 5 min elapsed, whichever occurred first. Appropriate discriminative performance for at least 2 training days (one preceded by 22-h deprivation, one preceded by 2-h deprivation) was required between generalization tests.

Immediately after generalization tests, subjects were placed in stainless steel cages and had access to a pre-weighed amount of regular food (~25 g of Teklad rat chow) placed on the floor of the cage, and water available in a bottle attached to the cage. Food intake was measured at the end of 1 h. Afterwards, rats were returned to their home cage and had free access to food and water until 2 h before the next training session.

##### Data analysis

One-way ANOVA was calculated (SPSS, Chicago, IL, USA) by assessing the effects of OT versus control conditions on the discriminative stimulus effects of 22-h food deprivation, lever pressing rate, and food intake. Tukey HSD *post hoc* tests were performed following significant ANOVA values to determine pairwise differences among conditions. Significance was set at *p* ≤ 0.05.

### Establishing OT-induced c-Fos Immunoreactivity in Feeding-Related Brain Sites in Rats Deprived for 2 and 22 h

For practical reasons, including the transfer of rats between cages and behavioral manipulations that could have affected baseline Fos expression, we chose a different cohort of animals here than those used in behavioral studies. Experimentally naive, age-matched male Sprague Dawley rats were divided into two cohorts (*n* = 12 per cohort) which were subjected to either 2 or 22 h of food deprivation (in both cases, deprivation period ended at 12:00). At the end of the deprivation, half of the animals in each cohort received an i.p. injection of isotonic saline, and the other half, 1 mg/kg OT. An hour after drug administration, animals were deeply anesthetized with urethane (35% dissolved in 0.9% saline, i.p.), and perfused through the aorta with 50 ml of saline followed by 500 ml of 4% paraformaldehyde in 0.1 phosphate buffer (pH 7.4). Brains were excised and post-fixed overnight in the same fixative at 4°C. 60 μm-thick coronal sections were cut with a vibratome (Leica, Germany) and later processed as free-floating sections for standard single antigen immunostaining of c-Fos.

#### Immunohistochemistry

Sections were rinsed in 50 nM TBS (pH 7.4–7.6), and then pre-treated for 10 min in 3% H_2_O_2_, 10% methanol (diluted in TBS). After rinsing in TBS they were incubated overnight at 4°C in the primary rabbit-anti-Fos antibody (diluted 1:3000; Synaptic Systems, Australia) washed in TBS, and subsequently incubated for 1 h at room temperature in the secondary goat-anti-rabbit antibody (1:400; Vector Laboratories). Following four washes in TBS, sections were incubated for 1 h with the avidin–biotin peroxidase complex (1:800; Elite Kit, Vector Laboratories). The vehicle for all incubations was a solution of 0.25% gelatin and 0.5% Triton X-100 in TBS. The peroxidase in the tissue was visualized with 0.05% diaminobenzidine (DAB), 0.01% H_2_O_2_ and 0.3% nickel sulfate (12-min incubation). Sections were washed four times in TBS to stop the reaction, mounted onto gelatin-coated slides, air-dried, dehydrated in ascending concentrations of ethanol, soaked in xylene (Merck KGaA, Germany) and embedded in Entellan (Merck KGaA, Germany). The number of Fos-positive nuclei per 1 mm^2^ was counted bilaterally for each neuroanatomical region of interest using ImageJ Software, with boundaries defined according to the Paxinos and Watson brain atlas, on 2–4 sections per animal. Images provided by a CCD camera attached to a Nikon Eclipse 400 microscope were analyzed using Nikon NIS Elements image software. The following areas were analyzed (in the parentheses, anterior-posterior ranges of bregma levels of sections used to analyze each site are shown): AcbC—nucleus accumbens core (1.28–0.96); AcbS—nucleus accumbens shell (1.28–0.96); AP—area postrema (−13.92 to −14.16); ARC—arcuate nucleus (−2.16 to −2.52); BLA—basolateral amygdala (−2.64 to −2.92); CEA—central nucleus of the amygdala (−2.64 to −2.92); DMH—dorsomedial nucleus of the hypothalamus (−3.00 to −3.24); DMV—dorsal motor nucleus of the vagus (−13.76 to −14.16); NTS—nucleus of the solitary tract (−13.76 to −14.16); PVN—paraventricular nucleus of the hypothalamus (−1.56 to −1.92); SON—supraoptic nucleus (−0.96 to −1.2); VMH—ventromedial nucleus (−3.00 to −3.24); VTA—ventral tegmental area (−6.72 to −6.84).

#### Data Analysis

Densities of Fos-positive nuclear profiles (per 1 mm^2^) were averaged per individual, and then per group. Data between the two groups (saline vs. OT) in each cohort were compared using a *t*-test. Values were considered significantly different for *p* ≤ 0.05.

### Establishing the Effect of Food Deprivation on Brainstem Expression of the OT Receptor Gene

#### Deprivation and Brain Stem Collection

The rats were divided to two groups. One group (*n* = 9) had unlimited access to standard chow and water, whereas the other had food taken away ~24 h before the animals were sacrificed by decapitation (*n* = 13). The brain stem was dissected and put in RNAlater (Ambion) for 2 h at room temperature and the samples were then frozen at −80C until further preparation.

#### rtPCR Protocol and Data Analysis

A standard protocol of sample preparation and rtPCR was used and, for brevity reasons, the main steps are described here [see (30) for details]. Samples were homogenized in TRIzol (Ambion); RNA was extracted with chloroform and precipitated in isopropanol. After centrifuging, the pellet was washed, air-dried, and dissolved in the DNase buffer (NEB). The samples were treated with RNase-free DNase I (Merck) and the absence of genomic DNA was established by PCR of a 5% template. 100 ng/μl genomic DNA served as a positive control, whereas MilliQ H_2_O as a negative one. The product was analyzed by electrophoresis. 5 μg RNA samples were diluted with MilliQ H_2_O. RNA was reverse-transcribed in the master mix (Promega; 20 μl). Samples were incubated for 1 h (37°C), followed by PCR to confirm cDNA synthesis. RtPCR reactions were performed in duplicates. Sample cDNA template (25 ng) was used per primer [OT receptor primer sequences: ttcttctgctgctctgctcgt (fwd) and tcatgctgaagatggctgaga (rev)]. Expression of three housekeeping genes (glyceraldehyde-3-phosphate- dehydrogenase, β-actin, and β-tubulin) was used to calculate normalization factors (GeNorm). Primer efficiencies were calculated with LinRegPCR (HFRC) and Ct values were corrected for differences in primer efficiencies. rtPCR results were analyzed with a Student's *t*-test. Values are presented as means ± S.E.M and they were deemed significantly different when *p* ≤ 0.05.

## Results

**Figure 1:**
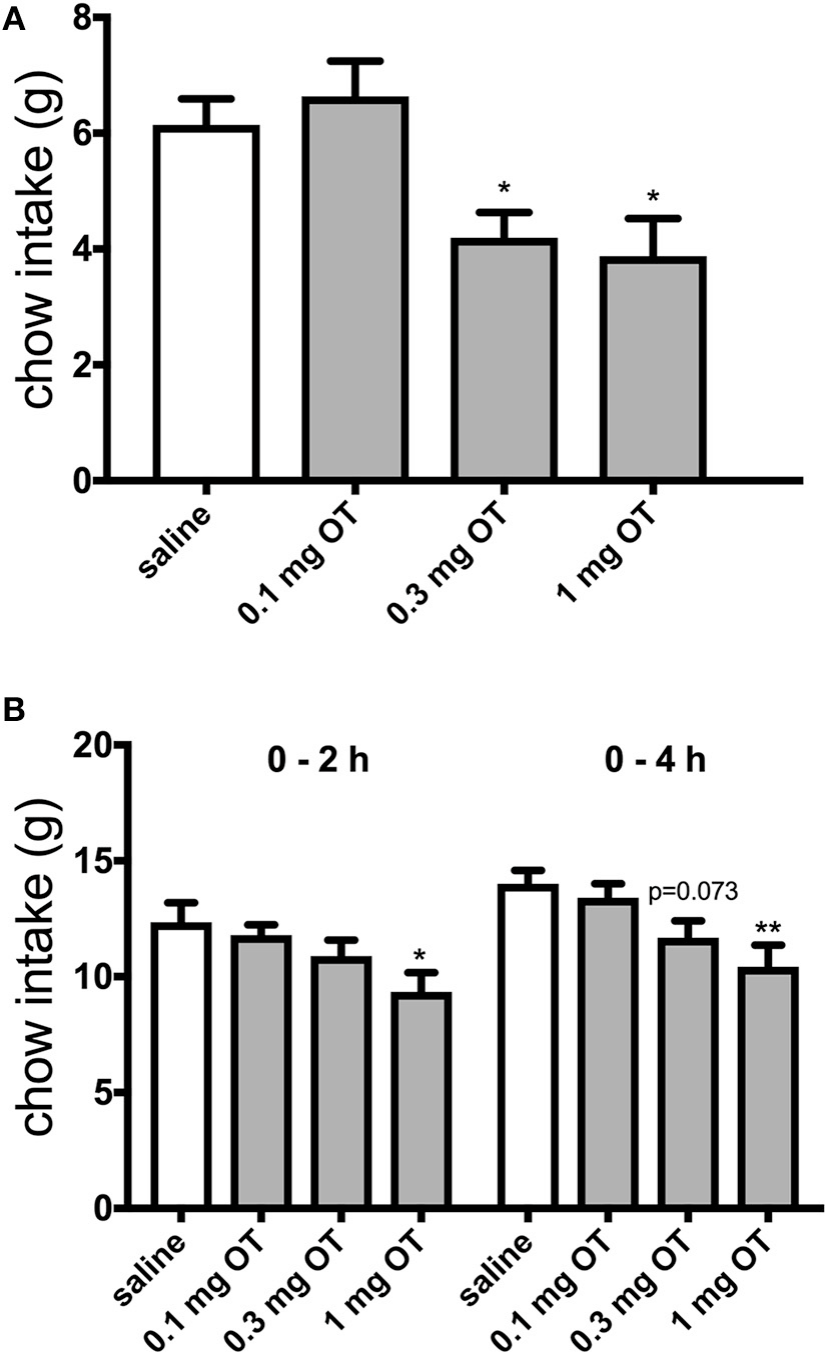
Effect of i.p. OT injection (0–1.0 mg/kg) on HFHS chow intake after a period of having no access to food for 2 h **(A)** or 22 h **(B)**. Saline was the vehicle. HFHS availability period was 2 h in the sated (2-h deprived) rats and 4 h in the 22-h deprived animals. Water was available ad libitum. OT at 0.3 mg and 1 mg/kg decreased HFHS food consumption [*F*_(3, 32)_ = 6.44; 0.3 mg, *p* = 0.042; 1 mg, *p* = 0.016] during the 2-h access period **(A)**. In animals subjected to 22-h food deprivation after which they gained access to the HFHS chow, 1 mg/kg OT decreased consumption at 2 h [*F*_(3, 32)_ = 3.38; *p* = 0.015] and 4 h [*F*_(3, 32)_ = 5.12; *p* = 0.004] of re-feeding **(B)**. There was a trend toward a decrease for 0.3 mg/kg OT at 4 h (*p* = 0.073). ^*^p < 0.05; ^**^p < 0.01.

The effects of 0.1, 0.3, and 1.0 mg/kg OT i.p. on HFHS palatable chow intake were investigated after 2 h and after 22 h of food deprivation. Control animals that had standard food taken away for 2 h and subsequently gained short-term access to the HFHS chow (thus, these rats were in effect sated) ate approximately 6 grams of the HFHS diet. OT at 0.3 mg/kg and 1 mg/kg decreased HFHS food consumption [*F*_(3, 32)_ = 6.44; 0.3 mg, *p* = 0.042; 1 mg, *p* = 0.016] during the 2-h access period by approximately 33% (Figure 1A). In animals subjected to 22-h food deprivation (which is a much more challenging energy deprivation scenario and it promotes search of and intake of caloric tastants) after which they gained access to the HFHS chow, 1 mg/kg OT decreased consumption by approximately 25% at 2 h [*F*_(3, 32)_ = 3.38; *p* = 0.015] and 4 h [*F*_(3, 32)_ = 5.12; *p* = 0.004) of re-feeding (Figure 1B). There was a trend toward a decrease for 0.3 mg/kg OT at 4 h (*p* = 0.073).

**Figure 2:**
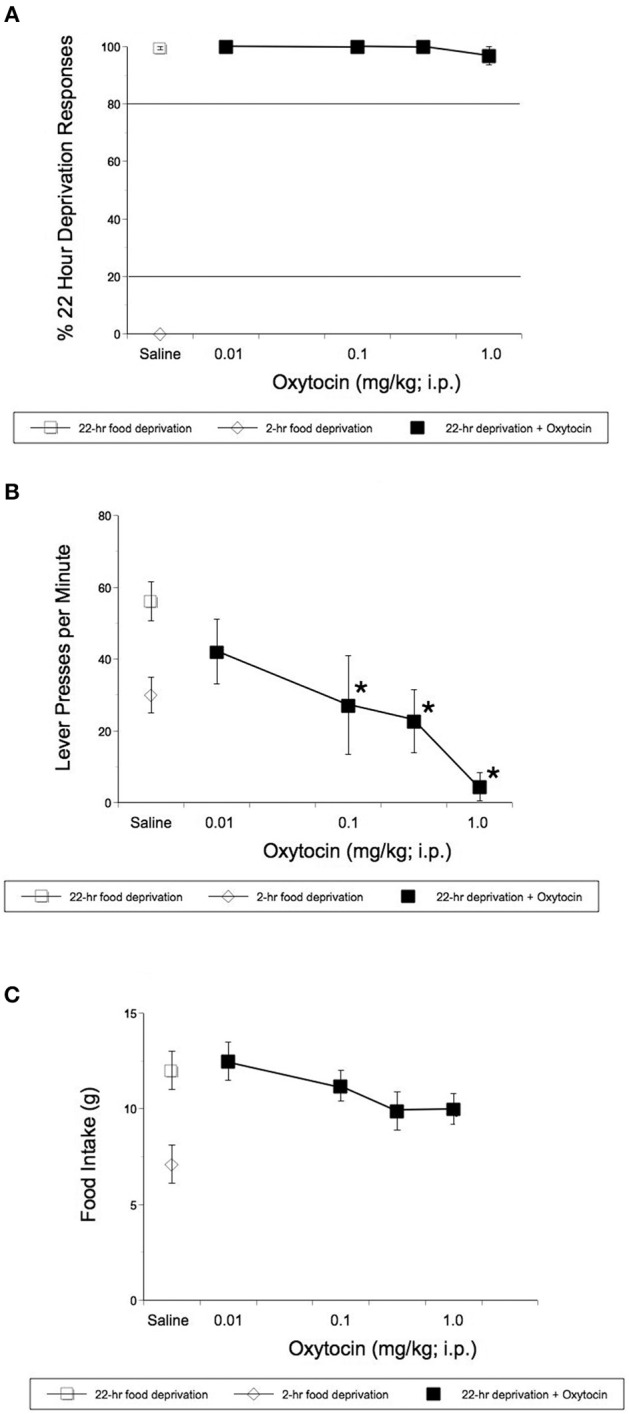
Effect of i.p. OT on the stimulus effects of 22-h food deprivation **(A)**, lever pressing response rates **(B)**, and regular laboratory chow intake in 1 h immediately following the completion of the discrimination test **(C)**. Animals had access to regular chow ad lib, before chow was withheld for 22 h and animals were injected. Saline was the vehicle. Operant studies revealed that OT did not alter the discriminative stimulus effects of 22-h food deprivation [*F*_(4, 16)_ = 1.00, *p* = 0.436 **(A)**]. OT did significantly alter response rates in rats [*F*_(4, 31)_ = 8.08, *p* = 0.0001 **(B)**]. OT significantly reduced rates of lever pressing following 0.1 (*p* = 0.044), 0.32 (*p* = 0.001), and 1 mg/kg OT i.p.(*p* < 0.0001) **(B)**. **(C)** Shows that animals deprived for 22 h and treated with OT, did not show significantly reduced consumption of regular chow in 1hr after the completion of the operant test. ^*^p < 0.05.

Rats learned to discriminate between 22- and 2-h food deprivation in a mean of 90 sessions. Operant studies revealed that OT even at doses that reduced HFHS diet intake in the experiments described above, did not alter the discriminative stimulus effects of 22-h food deprivation [*F*_(4, 16)_ = 1.00, *p* = 0.436, Figure 2A]. OT did significantly alter response rates in rats [*F*_(4, 31)_ = 8.08, *p* = 0.0001, Figure 2B]. OT significantly reduced rates of lever pressing following 0.1 mg/kg OT (p = 0.044), 0.32 (p = 0.001) and 1 mg/kg OT i.p.(*p* < 0.0001) (Figure 2B). As shown in Figure 2C, OT-treated animals deprived for 22 h and subjected to the hunger discrimination paradigm, did not show significantly reduced consumption of regular chow when they were transferred to a transition cage for 1 h after the operant test was concluded.

**Figure 3:**
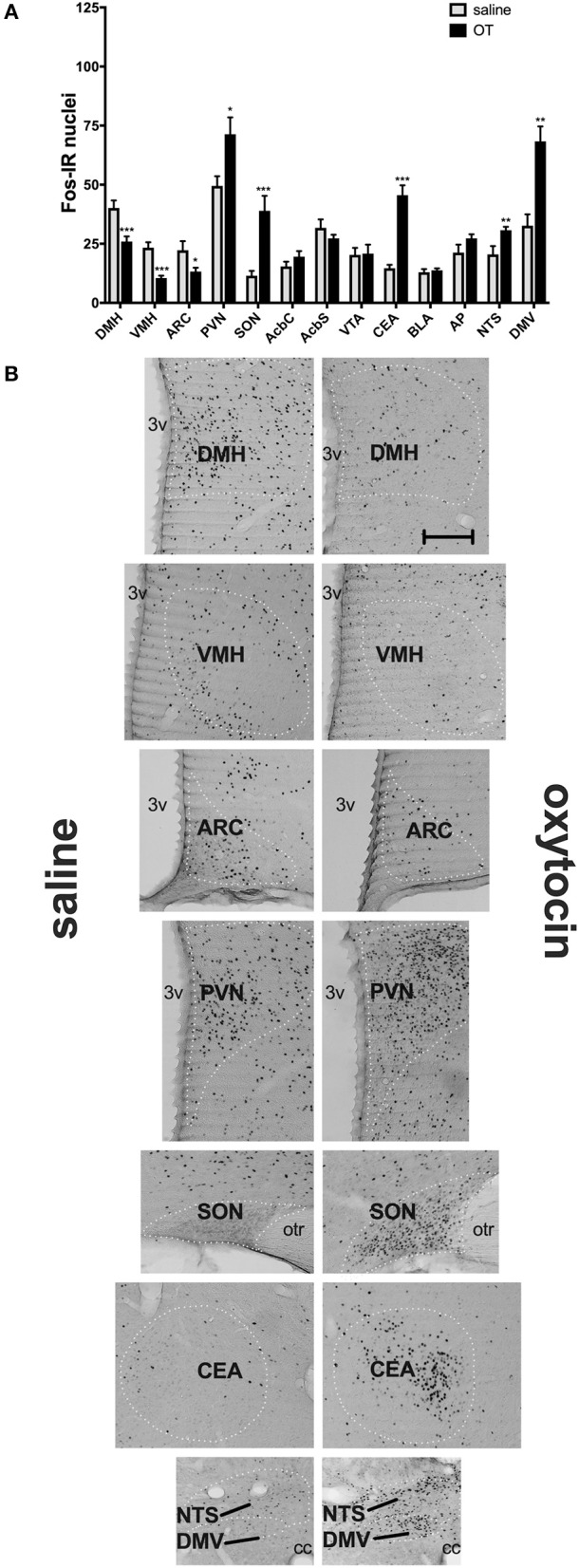
c-Fos immunoreactivity in feeding-related brain sites following i.p. administration of saline or OT (1 mg/kg) in animals that had no access to food for 2 h **(A)**. Panel **(B)** presents photomicrographs depicting sites that showed a significant difference in c-Fos levels (saline-treated rats—left side; OT-treated rats—right side). Densities of Fos-positive nuclear profiles (per 1 mm^2^ of a site) were averaged per individual, and then per group. AcbC, nucleus accumbens core; AcbS, nucleus accumbens shell; AP, area postrema; ARC, arcuate nucleus; BLA, basolateral amygdala; CEA, central nucleus of the amygdala; DMH, dorsomedial nucleus of the hypothalamus; DMV, dorsal motor nucleus of the vagus; NTS, nucleus of the solitary tract; PVN, paraventricular nucleus of the hypothalamus; SON, supraoptic nucleus; VMH, ventromedial nucleus; VTA, ventral tegmental area. ^*^p < 0.05; ^**^p < 0.01; ^***^p < 0.001.

**Figure 4:**
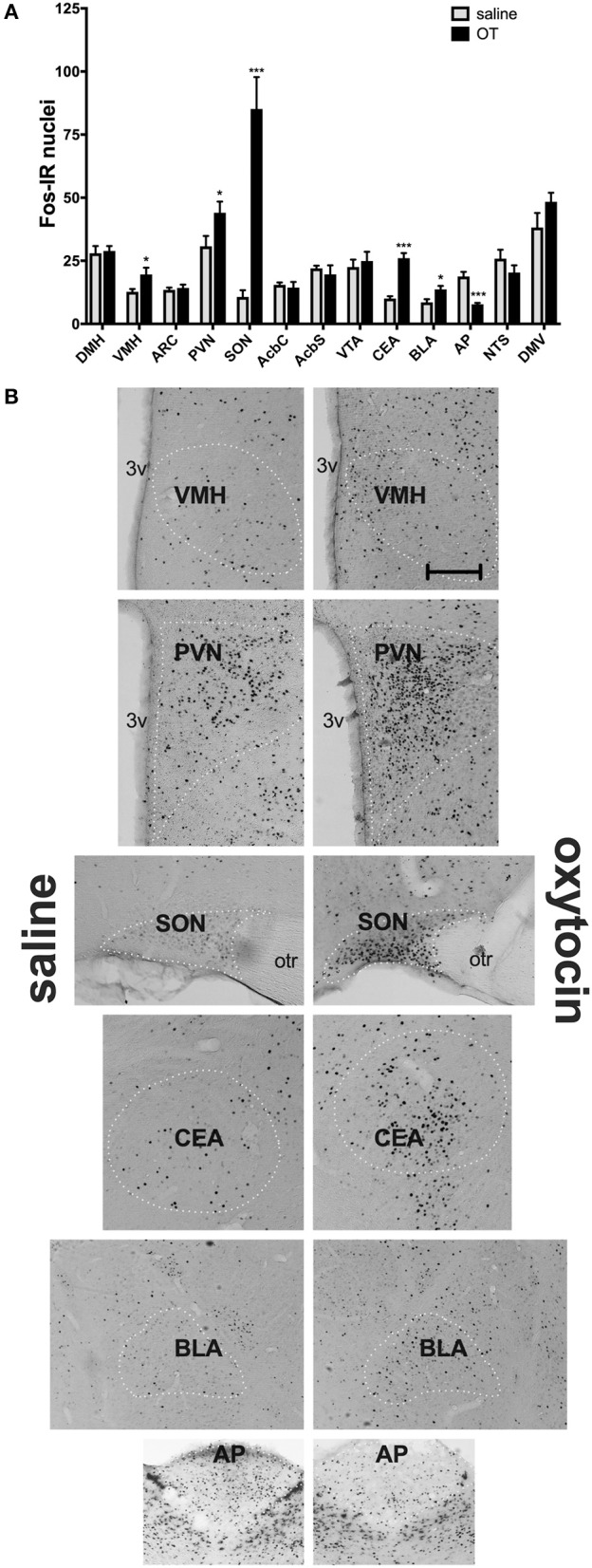
c-Fos immunoreactivity in feeding-related brain sites following i.p. administration of saline or OT (1 mg/kg) in animals that had no access to food for 22 h **(A)**. Panel **(B)** presents photomicrographs depicting sites that showed a significant difference in c-Fos levels (saline-treated rats—left side; OT-treated rats—right side). Densities of Fos-positive nuclear profiles (per 1 mm^2^ of a site) were averaged per individual, and then per group. AcbC, nucleus accumbens core; AcbS, nucleus accumbens shell; AP, area postrema; ARC, arcuate nucleus; BLA, basolateral amygdala; CEA, central nucleus of the amygdala; DMH, dorsomedial nucleus of the hypothalamus; DMV, dorsal motor nucleus of the vagus; NTS, nucleus of the solitary tract; PVN, paraventricular nucleus of the hypothalamus; SON, supraoptic nucleus; VMH, ventromedial nucleus; VTA, ventral tegmental area. ^*^p < 0.05; ^***^p < 0.001.

In a separate set of studies, we sought to investigate the effects of i.p. OT on neuronal activation in animals that are hungry and in animals that are sated. Intraperitoneal injection of OT at 1 mg/kg in rats deprived for 2 h affected c-Fos immunoreactivity in eight of the 13 feeding-related brain sites studied here (Figure 3). The number of c-Fos positive nuclei per mm^2^ in response to OT was elevated in the PVN (*p* = 0.011), SON (*p* < 0.001), NTS (*p* = 0.003), DMV (*p* < 0.001), and CEA (*p* < 0.001). A decrease was noted in the ARC (*p* = 0.019), VMH (*p* < 0.001), and DMH (*p* < 0.001). On the other hand, in animals deprived for 22 h (Figure 4), six areas showed differences in c-Fos levels: an increase was noted in the PVN (*p* = 0.042), SON (*p* < 0.001), VMH (*p* = 0.013), CEA (*p* < 0.001), and BLA (*p* = 0.012), whereas a decrease, in the AP (*p* < 0.001).

**Figure 5:**
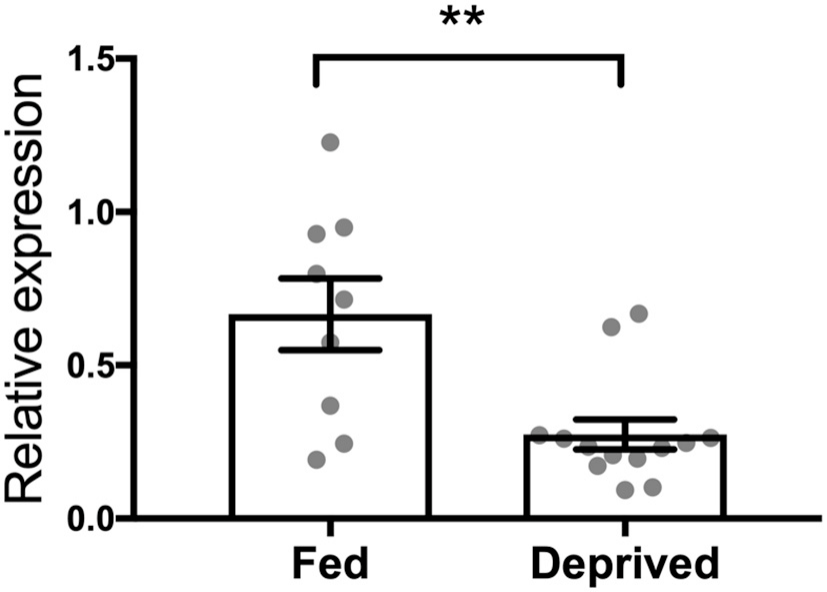
Effect of 24-h food deprivation on expression of the OT receptor gene established with real-time PCR in the brain stem. Ad libitum-fed rats served as controls. ^**^p < 0.01.

Finally, by applying rtPCR analysis, we showed a downregulation of the OT receptor gene in the brain stem of rats that underwent deprivation compared to their sated counterparts (*p* = 0.0024; Figure 5).

## Discussion

The fundamental drive that initiates food intake is a feeling of hunger. Hunger increases our motivation to seek high-energy foods and generates an avid feeding behavior upon encountering such ingestants. There are evolutionarily conserved neural and endocrine processes, such as those involving ghrelin or neuropeptide Y (NPY) (31–33), that promote eating for hunger, thereby ensuring that enough energy is obtained in order for the organism to maintain its functioning. However, in the obesogenic environment, where highly palatable and energy-rich diets are readily available, those mechanisms coupled with reward processes appear to be active even in the absence of actual energy needs, producing a feeling of hunger that leads to excessive food intake. Therefore, an important question that arises in the context of peptides that reduce consumption that can be potentially used clinically is whether they are capable of diminishing hunger responsiveness. Several decades of research on anorexigenic properties of OT have indicated that administration of OT supports early cessation of ingestive behavior [for review, see (34)]. Evidence gathered thus far has strongly linked OT with termination of feeding due to enhanced satiation or in response to adverse physiological changes (such as plasma osmolality or stomach distension) that endanger homeostasis. The current set of data shows for the first time that anorexigenic effects of OT do not stem from promoting a reduced feeling of hunger.

Previous experiments have shown beyond reasonable doubt that peripherally injected OT decreases consumption. It seems that OT is particularly effective in reducing intake of energy-dense solid foods regardless of their composition and/or palatability (2, 8, 13, 14). On the other hand, unlike central OT (particularly, targeting the VTA, AcbC or amygdala) or OT receptor ligands that cross the BBB, peripheral OT is not effective in modifying consumption of calorie-dilute sweet solutions that are ingested primarily for pleasure (3, 5, 10, 35). Our feeding experiments indicate that i.p. OT decreases calorie-dense palatable food consumption regardless of whether intake is stimulated predominantly by hunger (as in animals deprived for 22 h) or by palatability (as in animals that had the standard chow removed only 2 h prior to episodic access to the HFHS diet). Hence, the relative calorie needs do not impact the ability of OT to produce hypophagia. The effective dose range (0.3–1 mg/kg) is also similar regardless of a feeding paradigm used here and by others.

It should be noted that craving and motivation are not factors that are directly measurable in either our discrimination or consumption procedures. As we and others have reported, simple measures of food consumption do not necessarily relate to motivation to work to obtain food [e.g., (36, 37)]. Assuming there are only two motivating factors driving consumption (reward/pleasure or “free or hedonic” drives) and energy, the “free” or “hedonic” drives would be the key influence mediating consumption under the 2 h deprivation. In our experiments, OT was somewhat more potent in reducing HFHS consumption in subjects that were 2 h-deprived compared to subjects that were 22 h-deprived. OT (0.3 and 1.0 mg reduced HFHS consumption by approximately 33% in the former group. OT (0.3 mg) did not reduce consumption of the HFHS diet in the subjects food-restricted for 22 h. The reduction in intake under 22-h deprivation was about 25% suggesting that the contribution of the “free or hedonic” food intake may be at least to some extent reduced following the 22-h deprivation. We and others have shown that opioid antagonists are more potent in reducing consumption of sweet food compared to regular chow [for review, see e.g., (38, 39)]. These findings would also support the statement above. Craving is often associated with dependence-like behaviors during times of restriction or deprivation of the item inducing the “cravings.” We noted no dependence-like behaviors in subjects in either of our paradigms.

The hunger discrimination protocol that relies on the animals' ability to discriminate between short- (2–3 h) and long-term (e.g., 22 h) food deprivation and “report” it through operant behavior, has defined anorexigenic and orexigenic characteristics of several molecules. As food preloads appropriately change animals' perception of the length of deprivation, the parallel effect of anorexigens given instead of a food preload, indicates that they reduce a feeling of hunger. This approach allowed to implicate CCK and sibutramine as agents that can aid in controlling hunger (and deemed rimonabant ineffective in this process) (26, 27). Of note is the fact that, in contrast to anorexigens, some orexigens (NPY or ghrelin) make animals perceive the short 2-h deprivation as the state of hunger (28, 40). The fact that i.p. OT did not reduce operant responding to 22 h of deprivation strongly suggests that OT does not interfere with mechanisms that promote a feeling of hunger. It did not have an effect on hunger discrimination even though it was used at doses that are anorexigenic, and despite the fact that it did decrease bar pressing rate in general. It also produced a trend toward a reduction in chow intake in the 1 h after the completion of the operant test when animals were placed in a transition cage with chow present on the floor. Consequently, it can be inferred that OT induces hypophagia by being part of neuroendocrine processes that facilitate satiation and early cessation of feeding. This is in line with experiments showing a functional relationship between OT and numerous other mediators of satiety [for example, see (41, 42)], including melanocortins, which appear to form a common circuit with OT to support satiation. On the other hand, to our knowledge, there is no evidence that OT might be able to silence activation of, e.g., the NPY system, which could potentially suggest a link with hunger processing.

That i.p. OT differently affects neural processing under deprivation than in satiety is further substantiated by the outcomes of the c-Fos mapping study that revealed distinct patterns of c-Fos immunoreactivity in response to the OT treatment in hungry vs. sated animals. In individuals subjected to 2 h of deprivation, OT affected c-Fos immunoreactivity in a number of hypothalamic and brain stem sites: an increase in c-Fos staining occurred in the PVN, SON, NTS, and DMV, whereas a decrease was noted in the DMH, ARC, and VMH. The broad change in c-Fos levels in this network of sites that regulate energy intake is consistent with the role of OT as an anorexigen. Outside the hypothalamic-brainstem circuit, elevated c-Fos levels were in the CEA, which might potentially be related to emotional processing related to feeding (43–45) and it aligns well with the ability of OT itself to decrease consumption by acting at this site (5). Overall, the subset of sites activated by OT is quite similar to what had been reported before in various paradigms unrelated to food intake. For example, Carson et al. found an increase in the PVN, SON and CEA after 2 mg/kg OT in rats subjected to 80-min locomotor testing (46), and Hicks et al. found elevated activation in the PVN, SON, CEA and NTS after 1 mg/kg OT and a brief locomotor test (47). In another study, Hicks and colleagues reported an increase in the PVN, CEA, and NTS in adolescent animals (48).

In hungry (thus, 22-h deprived) rats, systemic OT induced c-Fos in a smaller subset of areas. In the hypothalamus and brain stem, the PVN, SON, VMH, and—unlike under 2-h deprivation—the AP showed a significant change (it should be noted though that the AP c-Fos levels in hungry animals treated with OT were higher too, though only a trend was observed). No difference was noted in the DMH, ARC, NTS, and DMV. It appears therefore that in the hungry state, OT loses the capacity to engage as broad a network of sites that regulate energy balance as that activated during satiety. rtPCR data showing a decreased expression of the OT receptor gene in the brain stem, a region thought to mediate anorexigenic properties of systemic OT, provide an additional insight into a mechanistic change that might be a key contributor to the changed, hunger/satiety-dependent receptivity of the CNS to OT. Of note is the fact that the rtPCR analysis revealed global brain stem expression changes and did not include individual areas. Considering that specific brain stem sites (and within these sites, specific neuronal populations) play distinct roles in appetite regulation, future studies will have to refine our understanding of a relationship between energy state, OT receptor, and these particular circuits within the brain stem. Finally, it should be noted that in both 22-h and 2-h deprived rats the amygdala was affected by OT, however in hungry animals, not only the CEA, but also the BLA expressed higher c-Fos immunoreactivity. It allows us to speculate that—considering the role of the amygdala in emotional processing (43–45)—OT affects emotional aspects of feeding regardless of the deprivation level.

We conclude that systemic OT does not diminish a feeling of hunger before the start of a meal. Instead, OT's anorexigenic properties can be manifested once consumption has already begun and this, at least to some extent, is driven by changes in brain responsiveness to OT treatment in the hungry vs. fed state. Therefore, OT's role in feeding control should be viewed as a mediator of early satiation rather than as a molecule that diminishes a perceived need to seek calories.

## Ethics Statement

This study was carried out in accordance with the recommendations of the University of Waikato animal ethics committee. The protocol was approved by the Institutional Animal Ethics Committee at the University of Waikato.

## Author Contributions

MH, DJ, SG, and AK performed the experiments. DJ, AL, and PO devised the project and the main conceptual ideas. MH, DJ, AK, PO, and SG processed and analyzed the data. MH, DJ, AL, and PO contributed to the organization and writing the manuscript.

### Conflict of Interest Statement

The authors declare that the research was conducted in the absence of any commercial or financial relationships that could be construed as a potential conflict of interest.

## References

[1] Arletti R Benelli A Bertolini A. Influence of oxytocin on feeding behavior in the rat. Peptides. (1989) 10:89–93. 10.1016/0196-9781(89)90082-X 2748428

[2] Blevins JE Thompson BW Anekonda VT Ho JM Graham JL Roberts ZS. Chronic CNS oxytocin signaling preferentially induces fat loss in high fat diet-fed rats by enhancing satiety responses and increasing lipid utilization. Am J Physiol Regul Integr Comp Physiol. (2016) 310:R640–58. 10.1152/ajpregu.00220.2015 26791828 PMC4867381

[3] Herisson FM Waas JR Fredriksson R Schioth HB Levine AS Olszewski PK. Oxytocin acting in the nucleus accumbens core decreases food intake. J Neuroendocrinol. (2016) 28. 10.1111/jne.12381 27114001

[4] Ho JM Anekonda VT Thompson BW Zhu M Curry RW Hwang BH. Hindbrain oxytocin receptors contribute to the effects of circulating oxytocin on food intake in male rats. Endocrinology. (2014) 155:2845–57. 10.1210/en.2014-1148 24877632 PMC4098005

[5] Klockars OA Klockars A Levine AS Olszewski PK. Oxytocin administration in the basolateral and central nuclei of amygdala moderately suppresses food intake. Neuroreport. (2018) 29:504–10. 10.1097/WNR.0000000000001005 29538098

[6] Klockars OA Waas JR Klockars A Levine AS Olszewski PK. Neural basis of ventromedial hypothalamic oxytocin-driven decrease in appetite. Neuroscience. (2017) 366:54–61. 10.1016/j.neuroscience.2017.10.008 29037599

[7] Olszewski PK Allen K Levine AS. Effect of oxytocin receptor blockade on appetite for sugar is modified by social context. Appetite. (2015) 86:81–7. 10.1016/j.appet.2014.10.007 25453587

[8] Roberts ZS Wolden-Hanson T Matsen ME Ryu V Vaughan CH Graham JL. Chronic hindbrain administration of oxytocin is sufficient to elicit weight loss in diet-induced obese rats. Am J Physiol Regul Integr Comp Physiol. (2017) 313:R357–71. 10.1152/ajpregu.00169.2017 28747407 PMC5668612

[9] Arletti R Benelli A Bertolini A. Oxytocin inhibits food and fluid intake in rats. Physiol Behav. (1990) 48:825–30. 10.1016/0031-9384(90)90234-U 2087513

[10] Melis MR Melis T Cocco C Succu S Sanna F Pillolla G. Oxytocin injected into the ventral tegmental area induces penile erection and increases extracellular dopamine in the nucleus accumbens and paraventricular nucleus of the hypothalamus of male rats. Eur J Neurosci. (2007) 26:1026–35. 10.1111/j.1460-9568.2007.05721.x 17672853

[11] Wu Z Xu Y Zhu Y Sutton AK Zhao R Lowell BB. An obligate role of oxytocin neurons in diet induced energy expenditure. PLoS ONE. (2012) 7:e45167. 10.1371/journal.pone.0045167 23028821 PMC3445456

[12] Maejima Y Iwasaki Y Yamahara Y Kodaira M Sedbazar U Yada T. Peripheral oxytocin treatment ameliorates obesity by reducing food intake and visceral fat mass. Aging. (2011) 3:1169–77. 10.18632/aging.100408 22184277 PMC3273897

[13] Morton GJ Thatcher BS Reidelberger RD Ogimoto K Wolden-Hanson T Baskin DG. Peripheral oxytocin suppresses food intake and causes weight loss in diet-induced obese rats. Am J Physiol Endocrinol Metab. (2012) 302:E134–44. 10.1152/ajpendo.00296.2011 22008455 PMC3328087

[14] Klockars A Brunton C Li L Levine AS Olszewski PK. Intravenous administration of oxytocin in rats acutely decreases deprivation-induced chow intake, but it fails to affect consumption of palatable solutions. Peptides. (2017) 93:13–9. 10.1016/j.peptides.2017.04.010 28460894

[15] Mitra A Gosnell BA Schioth HB Grace MK Klockars A Olszewski PK. Chronic sugar intake dampens feeding-related activity of neurons synthesizing a satiety mediator, oxytocin. Peptides. (2010) 31:1346–52. 10.1016/j.peptides.2010.04.005 20399242 PMC3175817

[16] Kublaoui BM Gemelli T Tolson KP Wang Y Zinn AR. Oxytocin deficiency mediates hyperphagic obesity of Sim1 haploinsufficient mice. Mol Endocrinol. (2008) 22:1723–34. 10.1210/me.2008-0067 18451093 PMC2453606

[17] Verbalis JG McCann MJ McHale CM Stricker EM. Oxytocin secretion in response to cholecystokinin and food: differentiation of nausea from satiety. Science. (1986) 232:1417–9. 10.1126/science.3715453 3715453

[18] Ohlsson B Forsling ML Rehfeld JF Sjolund K. Cholecystokinin stimulation leads to increased oxytocin secretion in women. Eur J Surg. (2002) 168:114–8. 10.1080/11024150252884340 12113268

[19] Renaud LP Tang M McCann MJ Stricker EM Verbalis JG. Cholecystokinin and gastric distension activate oxytocinergic cells in rat hypothalamus. Am J Physiol. (1987) 253(4 Pt 2):R661–5. 10.1152/ajpregu.1987.253.4.R661 3661761

[20] Bojanowska E Stempniak B. tGLP-1 and release of vasopressin and oxytocin from the isolated rat hypothalamo-neurohypophysial system: effects of a tGLP-1 receptor agonist and antagonist. J Physiol Pharmacol. (2001) 52(4 Pt 2):781–93. 11785773

[21] Ladyman SR Augustine RA Scherf E Phillipps HR Brown CH Grattan DR. Attenuated hypothalamic responses to alpha-melanocyte stimulating hormone during pregnancy in the rat. J Physiol. (2016) 594:1087–101. 10.1113/JP271605 26613967 PMC4753265

[22] Gartner SN Aidney F Klockars A Prosser C Carpenter EA Isgrove K. Intragastric preloads of l-tryptophan reduce ingestive behavior via oxytocinergic neural mechanisms in male mice. Appetite. (2018) 125:278–86. 10.1016/j.appet.2018.02.015 29471071

[23] Gartner SN Klockars A Prosser C Carpenter EA Levine AS Olszewski PK. Identification of central mechanisms underlying anorexigenic effects of intraperitoneal L-tryptophan. Neuroreport. (2018) 29:1293–300. 10.1097/WNR.0000000000001110 30085976

[24] Lawson EA Marengi DA DeSanti RL Holmes TM Schoenfeld DA Tolley CJ. Oxytocin reduces caloric intake in men. Obesity. (2015) 23:950–6. 10.1002/oby.21069 25865294 PMC4414748

[25] Olson BR Drutarosky MD Chow MS Hruby VJ Stricker EM Verbalis JG. Oxytocin and an oxytocin agonist administered centrally decrease food intake in rats. Peptides. (1991) 12:113–8. 10.1016/0196-9781(91)90176-P 1646995

[26] Corwin RL Woolverton WL Schuster CR Effects of cholecystokinin, d-amphetamine and fenfluramine in rats trained to discriminate 3 from 22 h of food deprivation. J Pharmacol Exp Therapeut. (1990) 253:720–8. 2338655

[27] Jewett DC Hahn TW Smith TR Fiksdal BL Wiebelhaus JM Dunbar AR. Effects of sibutramine and rimonabant in rats trained to discriminate between 22- and 2-h food deprivation. Psychopharmacology. (2009) 203:453–9. 10.1007/s00213-008-1350-1 18854986

[28] Jewett DC Lefever TW Flashinski DP Koffarnus MN Cameron CR Hehli DJ. Intraparaventricular neuropeptide Y and ghrelin induce learned behaviors that report food deprivation in rats. Neuroreport. (2006) 17:733–7. 10.1097/01.wnr.0000215767.94528.fb 16641678

[29] Olszewski PK Klockars A Olszewska AM Fredriksson R Schioth HB Levine AS Molecular, immunohistochemical, and pharmacological evidence of oxytocin's role as inhibitor of carbohydrate but not fat intake. Endocrinology. (2010) 151:4736–44. 10.1210/en.2010-0151 20685878 PMC2946140

[30] Fredriksson R Hagglund M Olszewski PK Stephansson O Jacobsson JA Olszewska AM. The obesity gene, FTO, is of ancient origin, up-regulated during food deprivation and expressed in neurons of feeding-related nuclei of the brain. Endocrinology. (2008) 149:2062–71. 10.1210/en.2007-1457 18218688

[31] Stanley BG Kyrkouli SE Lampert S Leibowitz SF. Neuropeptide Y chronically injected into the hypothalamus: a powerful neurochemical inducer of hyperphagia and obesity. Peptides. (1986) 7:1189–92. 10.1016/0196-9781(86)90149-X 3470711

[32] Bailey AR Giles M Brown CH Bull PM Macdonald LP Smith LC. Chronic central infusion of growth hormone secretagogues: effects on fos expression and peptide gene expression in the rat arcuate nucleus. Neuroendocrinology. (1999) 70:83–92. 10.1159/000054462 10461022

[33] Wren AM Small CJ Abbott CR Dhillo WS Seal LJ Cohen MA. Ghrelin causes hyperphagia and obesity in rats. Diabetes. (2001) 50:2540–7. 10.2337/diabetes.50.11.2540 11679432

[34] Olszewski PK Klockars A Levine AS. Oxytocin: a conditional anorexigen whose effects on appetite depend on the physiological, behavioural and social contexts. J Neuroendocrinol. (2016) 28. 10.1111/jne.12376 26918919

[35] Herisson FM Brooks LL Waas JR Levine AS Olszewski PK. Functional relationship between oxytocin and appetite for carbohydrates versus saccharin. Neuroreport. (2014) 25:909–14. 10.1097/WNR.0000000000000201 24893201

[36] Jewett DC Cleary J Levine AS Schaal DW Thompson T. Effects of neuropeptide Y on food-reinforced behavior in satiated rats. Pharmacol Biochem Behav. (1992) 42:207–12. 10.1016/0091-3057(92)90517-J 1631179

[37] Jewett DC Cleary J Levine AS Schaal DW Thompson T. Effects of neuropeptide Y, insulin, 2-deoxyglucose, and food deprivation on food-motivated behavior. Psychopharmacology. (1995) 120:267–71. 10.1007/BF02311173 8524973

[38] Olszewski PK Wood EL Klockars A Levine AS. Excessive consumption of sugar: an insatiable drive for reward. Curr Nutr Rep. (2019) 8:120–8. 10.1007/s13668-019-0270-5 30945139

[39] Olszewski PK Levine AS. Central opioids and consumption of sweet tastants: when reward outweighs homeostasis. Physiol Behav. (2007) 91:506–12. 10.1016/j.physbeh.2007.01.011 17316713

[40] Davidson TL Kanoski SE Tracy AL Walls EK Clegg D Benoit SC. The interoceptive cue properties of ghrelin generalize to cues produced by food deprivation. Peptides. (2005) 26:1602–10. 10.1016/j.peptides.2005.02.014 16112399

[41] Olson BR Drutarosky MD Stricker EM Verbalis JG. Brain oxytocin receptor antagonism blunts the effects of anorexigenic treatments in rats: evidence for central oxytocin inhibition of food intake. Endocrinology. (1991) 129:785–91. 10.1210/endo-129-2-785 1649746

[42] Olszewski PK Wirth MM Shaw TJ Grace MK Billington CJ Giraudo SQ. Role of alpha-MSH in the regulation of consummatory behavior: immunohistochemical evidence. Am J Physiol Regul Integr Comp Physiol. (2001) 281:R673–80. 10.1152/ajpregu.2001.281.2.R673 11448874

[43] Sun X Kroemer NB Veldhuizen MG Babbs AE de Araujo IE Gitelman DR. Basolateral amygdala response to food cues in the absence of hunger is associated with weight gain susceptibility. J Neurosci. (2015) 35:7964–76. 10.1523/JNEUROSCI.3884-14.2015 25995480 PMC4438134

[44] Beckman TR Shi Q Levine AS Billington CJ. Amygdalar opioids modulate hypothalamic melanocortin-induced anorexia. Physiol Behav. (2009) 96:568–73. 10.1016/j.physbeh.2008.12.007 19136019 PMC4284077

[45] Alvarez-Crespo M Skibicka KP Farkas I Molnar CS Egecioglu E Hrabovszky E. The amygdala as a neurobiological target for ghrelin in rats: neuroanatomical, electrophysiological and behavioral evidence. PLoS ONE. (2012) 7:e46321. 10.1371/journal.pone.0046321 23071554 PMC3468604

[46] Carson DS Hunt GE Guastella AJ Barber L Cornish JL Arnold JC. Systemically administered oxytocin decreases methamphetamine activation of the subthalamic nucleus and accumbens core and stimulates oxytocinergic neurons in the hypothalamus. Addict Biol. (2010) 15:448–63. 10.1111/j.1369-1600.2010.00247.x 20731630

[47] Hicks C Ramos L Dampney B Baracz SJ McGregor IS Hunt GE. Regional c-Fos expression induced by peripheral oxytocin administration is prevented by the vasopressin 1A receptor antagonist SR49059. Brain Res Bull. (2016) 127:208–18. 10.1016/j.brainresbull.2016.10.005 27725169

[48] Hicks C Jorgensen W Brown C Fardell J Koehbach J Gruber CW. The nonpeptide oxytocin receptor agonist WAY 267,464: receptor-binding profile, prosocial effects and distribution of c-Fos expression in adolescent rats. J Neuroendocrinol. (2012) 24:1012–29. 10.1111/j.1365-2826.2012.02311.x 22420322 PMC3399775

